# Psilocybin for the treatment of Alzheimer’s disease

**DOI:** 10.3389/fnins.2024.1420601

**Published:** 2024-07-10

**Authors:** Siyi Zheng, Rong Ma, Yang Yang, Gang Li

**Affiliations:** ^1^Department of Neurology, Union Hospital, Tongji Medical College, Huazhong University of Science and Technology, Wuhan, China; ^2^Department of Pharmacology, School of Basic Medicine, Tongji Medical College, Huazhong University of Science and Technology, Wuhan, China; ^3^Department of General Medicine, Binzhou Medical University Hospital, Binzhou, China

**Keywords:** psilocybin, Alzheimer’s disease, 5-HT2A receptor, neuroplasticity, anti-inflammation, pharmacology

## Abstract

Alzheimer’s disease (AD) stands as a formidable neurodegenerative ailment and a prominent contributor to dementia. The scarcity of available therapies for AD accentuates the exigency for innovative treatment modalities. Psilocybin, a psychoactive alkaloid intrinsic to hallucinogenic mushrooms, has garnered attention within the neuropsychiatric realm due to its established safety and efficacy in treating depression. Nonetheless, its potential as a therapeutic avenue for AD remains largely uncharted. This comprehensive review endeavors to encapsulate the pharmacological effects of psilocybin while elucidating the existing evidence concerning its potential mechanisms contributing to a positive impact on AD. Specifically, the active metabolite of psilocybin, psilocin, elicits its effects through the modulation of the 5-hydroxytryptamine 2A receptor (5-HT2A receptor). This modulation causes heightened neural plasticity, diminished inflammation, and improvements in cognitive functions such as creativity, cognitive flexibility, and emotional facial recognition. Noteworthy is psilocybin’s promising role in mitigating anxiety and depression symptoms in AD patients. Acknowledging the attendant adverse reactions, we proffer strategies aimed at tempering or mitigating its hallucinogenic effects. Moreover, we broach the ethical and legal dimensions inherent in psilocybin’s exploration for AD treatment. By traversing these avenues, We propose therapeutic potential of psilocybin in the nuanced management of Alzheimer’s disease.

## 1 Introduction

Alzheimer’s disease (AD) is a progressive neurodegenerative disease that is the leading cause of dementia in the elderly population (Anonymous, 2021). It is characterized by the deposition of amyloid-beta (Aβ) plaques, tau neurofibrillary tangles, and neuroinflammation (Scheltens et al., 2021). The prevalence of dementia is expected to rise as the global population grows and ages, with projections estimating a significant increase in the number of cases (Anonymous, 2022b). In 2019, the total cost of healthcare, long-term care, and hospice services for individuals aged 65 years and older with dementia in the United States was estimated at $2.2billion, so AD imposes a substantial burden on individuals, families, society, and the economy (Anonymous, 2022a). The U.S. Food and Drug Administration (FDA) has approved seven drugs for the treatment of AD, including rivastigmine, donepezil, galantamine, memantine, memantine combined with donepezil, aducanumab and lecanemab. Among them, aducanumab and lecanemab are part of the first wave of Aβ-targeting drugs for AD and are solely recommended for early-stage AD (Anonymous, 2021, 2022b; van Dyck et al., 2023). It is important to highlight that these medications are linked to a significant incidence of adverse reactions, including amyloid-related imaging abnormalities, brain edema, and headaches (Salloway et al., 2022; van Dyck et al., 2023). Furthermore, they impose a considerable financial burden due to their high cost (Glymour et al., 2022; Burke et al., 2023). Given the significant burden of AD, it is crucial to continue developing new therapies to effectively treat the disease.

In recent years, research on the use of psychedelics in the field of mental and nervous systems has garnered widespread attention and enthusiasm (Roth and Gumpper, 2023; Siegel et al., 2023). Among them, psilocybin, a naturally occurring hallucinogenic compound found in certain species of mushrooms, particularly those belonging to the genus *Psilocybe*, has garnered increasing attention (Lenz et al., 2020). Psilocybin has shown promise as a treatment for major depressive disorder (MDD), and in 2019, psilocybin therapy was designated as a breakthrough therapy by the FDA (Donovan et al., 2021; Raison et al., 2023). In the Phase 2 double-blind trial conducted by COMPASS, administering a dosage of 25 mg of psilocybin for the treatment of treatment-resistant depression (TRD) demonstrated a significant reduction in the Montgomery-Åsberg Depression Rating Scale scores of the subjects (Goodwin et al., 2022). Furthermore, several small-scale clinical trials have indicated the potential benefits of psilocybin for a range of conditions, such as anxiety disorders (Griffiths et al., 2016; Ross et al., 2016), obsessive-compulsive disorder (Moreno et al., 2006; Ching et al., 2023), migraine (Schindler et al., 2021), cluster headache (Schindler et al., 2022), addictive behaviors (Bogenschutz et al., 2022; Slomski, 2022), body dysmorphic disorder (Schneier et al., 2023), as well as anorexia nervosa (Peck et al., 2023).

Although the use of psilocybin as a treatment for AD has not been sufficiently studied, there is still a potential for psilocybin to be explored as a new treatment option in the future. The density of 5-HT2A receptor is reduced in AD, and this reduction is associated with a decline in cognitive function (Versijpt et al., 2003). Studies on AD rats induced with streptozotocin have shown that the administration of 5-HT1A and 5-HT2A receptor agonists has significant neuroprotective effects on hippocampal neurons through anti-apoptotic and anti-inflammatory pathways (Shahidi et al., 2019). Psilocybin, known for its mechanisms in promoting neuroplasticity, anti-inflammation, and improving functional connectivity of brain networks in the treatment of depression and other diseases, may hold potential benefits for patients with AD (Nkadimeng et al., 2021; Daws et al., 2022; Du et al., 2023). Additionally, depression and anxiety are common mental symptoms of AD and can accelerate cognitive decline in AD patients (Gallagher et al., 2018; Ma, 2020; Agüera-Ortiz et al., 2021). Psilocybin may potentially delay the progression of AD by alleviating depression and anxiety symptoms.

**Figure 1:**
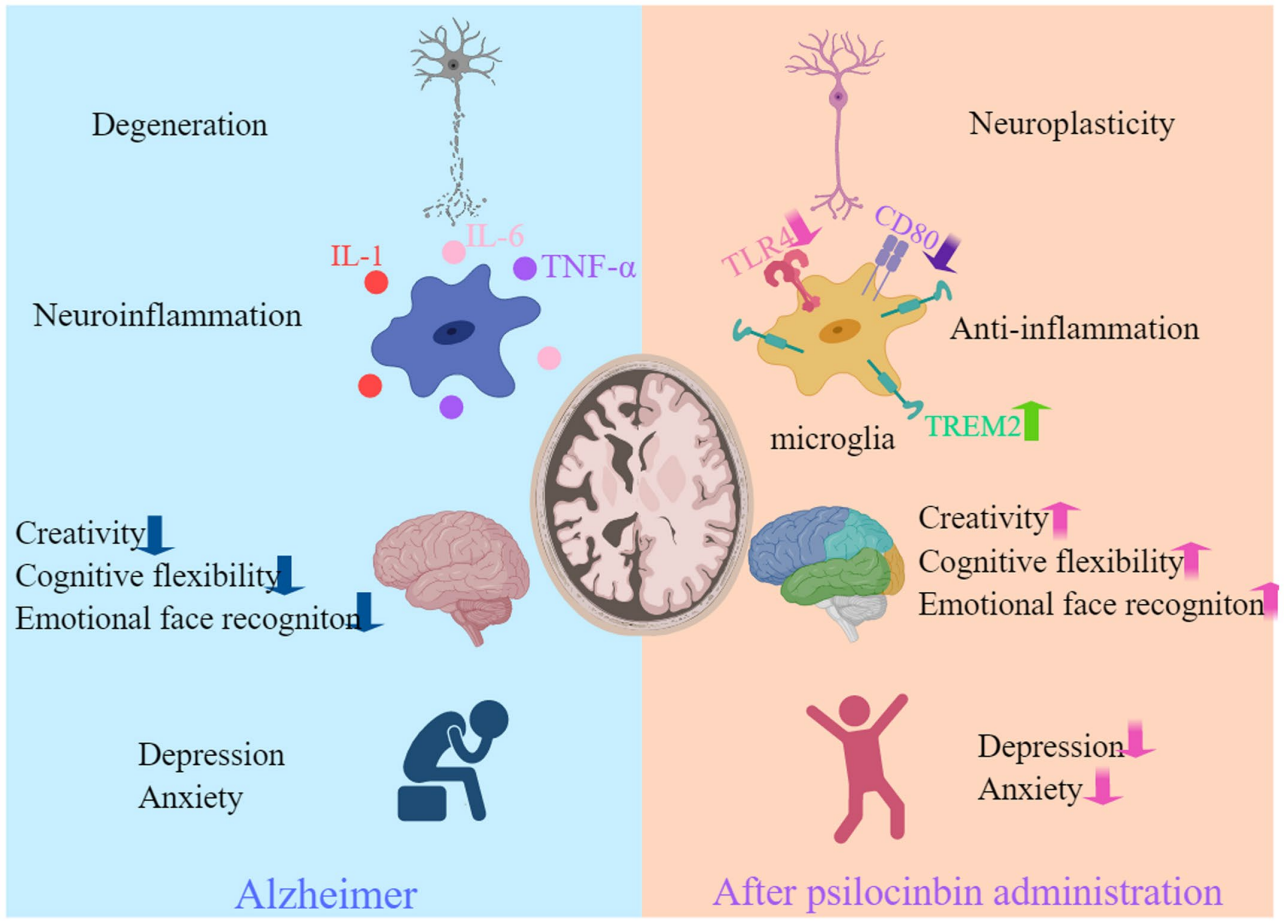
Psilocybin may potentially benefit individuals with AD. Psilocybin has shown benefits for AD in terms of enhancing neuroplasticity, reducing inflammation, neurocognitive improvement, and alleviating anxiety and depression. Created with MedPeer (www.medpeer.cn).

While recent reviews have broached the potential of hallucinogens in treating neurodegenerative diseases (Vann Jones and O’Kelly, 2020; Garcia-Romeu et al., 2022; Kozlowska et al., 2022; Pilozzi et al., 2023), this review uniquely focuses on psilocybin and its application in AD. Introducing the latest research findings and clinical trials, this review provides an in-depth exploration of the pharmacology of psilocybin and the potential mechanisms underlying its therapeutic effects in AD (Figure 1). This review shines a spotlight on its thorough amalgamation of the adverse effects of psilocybin and the tactics employed to alleviate its hallucinogenic qualities, ultimately facilitating the process of clinical translation. Lastly, as the landscape of psilocybin therapy evolves, attention is drawn to the concurrent emergence of legal and ethical considerations.

## 2 Pharmacological effects of psilocybin

### 2.1 Metabolic processes

**Figure 2:**
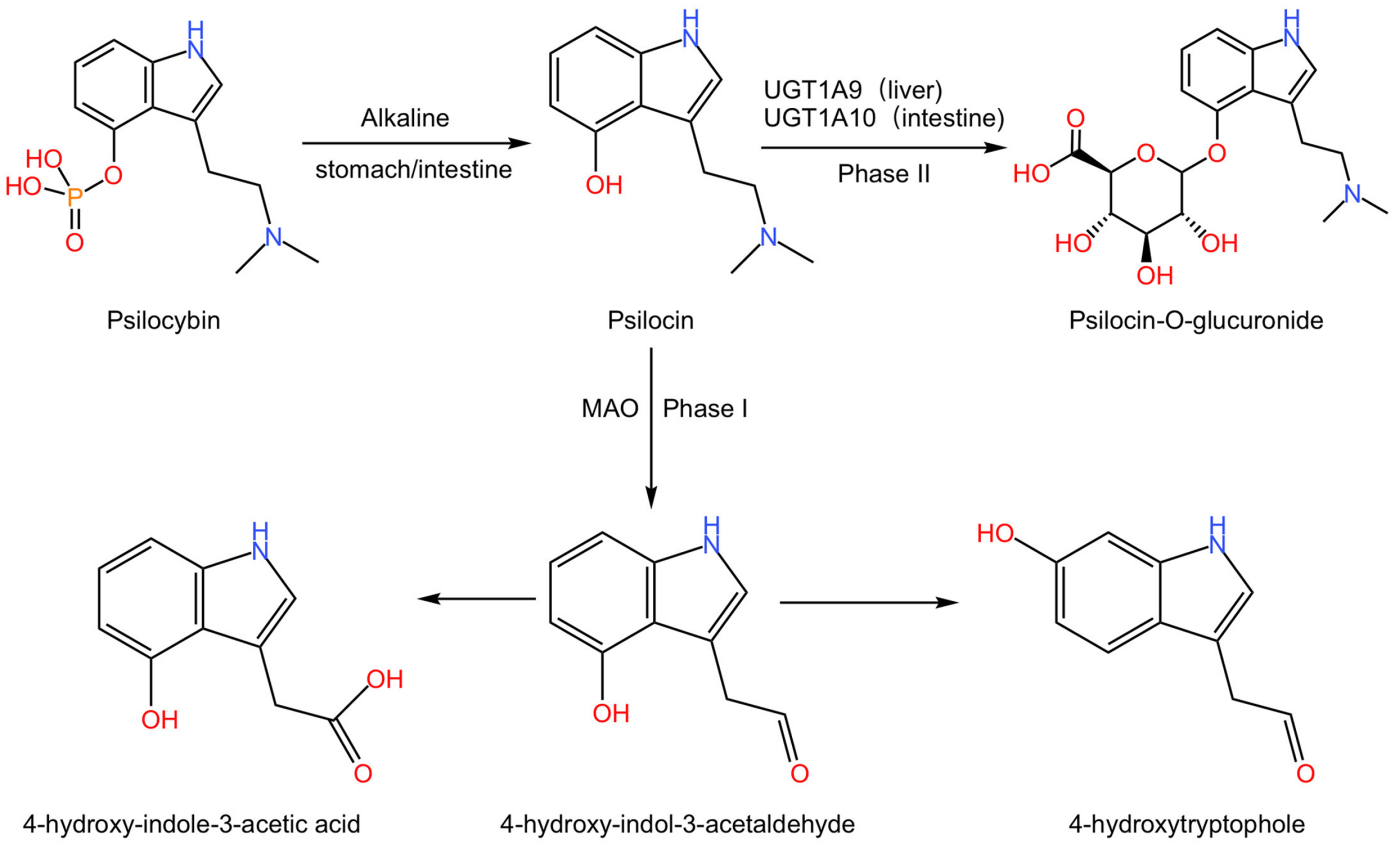
The metabolism process of psilocybin. Psilocybin undergoes dephosphorylation under the alkaline conditions of the stomach or intestine to form psilocin. In phase I, psilocin undergoes MAO action to generate 4-hydroxyindole-3-acetaldehyde, which further oxidizes to produce 4-hydroxy-indole-3-acetic acid or reduces to 4-hydroxytryptophole. And in phase II, psilocin is converted to psilocin-O-glucuronide by UGT1A9 in the liver or UGT1A10 in the intestine.

The metabolic pathways of psilocybin have been confirmed in healthy volunteers. After oral administration, psilocybin undergoes dephosphorylation by alkaline phosphatase in the stomach and intestine to form psilocin, which can then cross the blood–brain barrier and exert its hallucinogenic effects and related pharmacological effects (Horita and Weber, 1961a,b, 1962). Psilocin has two major metabolic pathways, phase I and phase II. The phase II metabolic pathway is the primary pathway, accounting for ≥80% of the total metabolism (Manevski et al., 2010). In the phase I metabolic pathway, psilocin undergoes oxidative reactions via monoamine oxidase (MAO) in the liver, resulting in the formation of 4-hydroxyindole-3-acetaldehyde, which further undergoes oxidation to form 4-hydroxy-indole-3-acetic acid, or reduction to form 4-hydroxytryptophole (Kalberer et al., 1962; Lindenblatt et al., 1998; Kolaczynska et al., 2021). In the phase II metabolic pathway, psilocin undergoes enzymatic catalysis by UDP-glucuronosyltransferases (UGT) 1A10 in the intestine and UGT1A9 in the liver to form O-glucuronide conjugates, which are then excreted in the urine (Hasler et al., 2002; Kamata et al., 2006; Manevski et al., 2010; Figure 2). Psilocin reaches its peak plasma concentration approximately 2–3 h after administration and has a half-life of about 3 h (standard deviation 1.1) (Brown et al., 2017; Becker et al., 2022).

The majority of clinical research has utilized oral administration, resulting in limited data on intravenous metabolism. After intravenous administration of 1 mg in healthy volunteers, a mean psilocin maximum plasma concentration of 12.9 ± 5.6 ng/mL plasma was observed 1.9 ± 1.0 min after intravenous injection. 4-hydroxyindole-3-acetic acid was not detected following the intravenous psilocin administration (Hasler et al., 1997).

### 2.2 5-HT receptors

The mechanism of action of psilocin remains intricate and not fully elucidated. Psilocin engages with a myriad of 5-HT receptors, encompassing 5-HT1A receptor, 5-HT1B receptor, 5-HT1D receptor, 5-HT2A receptor, 5-HT2B receptor, 5-HT2C receptor, 5-HT5 receptor, 5-HT6 receptor, and 5-HT7 receptor. This information is documented in the Psychoactive Drug Screening Program database.^1^

Of these, the 5-HT2A receptor stands out as the principal mediator of psilocin’s hallucinogenic effects. The 5-HT2A receptor antagonist ketanserin has proven efficacy in inhibiting psilocin-induced perceptual distortions in humans and the head twitching reaction in mice, a behavioral characteristic strongly associated with hallucinations (Vollenweider et al., 1998; Madsen et al., 2019; Hesselgrave et al., 2021).

Beyond the 5-HT2A receptor, the 5-HT1A receptor, either directly or through functional interactions with the 5-HT2A receptor, modulates psilocin-induced hallucinations. Administration of the partial agonist buspirone, targeting the 5-HT1A receptor, effectively alleviates subjective symptoms associated with psilocybin use in humans (Pokorny et al., 2016). Activation of 5-HT1A receptor and 5-HT2A receptor manifests opposing effects, with 5-HT2A receptor activation being dominant in the presence of psilocin. Specifically, activation of the 5-HT2A receptor leads to pyramidal neuron excitation, while activation of the 5-HT1A receptor inhibits their activity (Preller et al., 2020). Moreover, changes in global brain connectivity induced by psilocin exhibit a strong positive correlation with the expression of the 5-HT2A receptor gene, but a negative correlation with the expression of the 5-HT1A receptor gene (Preller et al., 2020). Administration of the 5-HT2C receptor antagonist RS-102221 demonstrates a dose-dependent modulation of 5-hydroxytryptamine receptor activity, wherein lower doses are associated with augmentation of HTR, whereas higher doses result in a decrement in HTR (Shahar et al., 2022).

The ongoing discourse revolves around the crucial inquiry into whether the antidepressant efficacy of psilocin is contingent upon the activation of the 5-HT2A receptor. Contrasting findings exist; certain investigations propose that ketanserin’s incapacity to impede the antidepressant effects and dendritic spine formation in chronically stressed mice induced by psilocybin implies a potential dissociation from 5-HT2A receptor blockade (Hesselgrave et al., 2021; Shao et al., 2021). The prospect that the 5-HT2A receptor mediates these effects warrants careful consideration. This discordance may arise from various factors: a potential inadequacy in ketanserin dosage to effectively obstruct the 5-HT2A receptor, direct initiation of the brain-derived neurotrophic factor-tropomyosin receptor kinase B (BDNF–TRKB) signaling pathway downstream of 5-HT2A receptor by psilocin, or the plausible involvement of other 5-HT receptors in facilitating the antidepressant effects. The augmentation of neuroplasticity and enhancement of neural connectivity through the activation of intracellular 5-HT2A receptor by psilocin substantiate the critical role of 5-HT2A receptor activation in achieving sustained antidepressant outcomes (Vargas et al., 2023). The proposition to generate 5-HT2A receptor knockout mice could potentially elucidate the necessity of 5-HT2A receptor activation in the context of psilocybin’s effects (Table 1).

**Table 1:** Summary of clinical trials on psilocybin.

References	Size	Subjects	Dosage	Assessment time/follow-up duration	Outcomes
Rosenblat et al. (2024)	30	MDD/bipolar II disorder	25 mg, 3 doses	2 weeks	Repeated doses were associated with greater reductions in depression severity.
Pagni et al. (2024)	11	Alcohol use disorder (AUD)	A single 25 mg dose	2 days	Psilocybin induced changes in neural reactivity to alcohol and emotional cues.
Agrawal et al. (2024)	30	Cancer patients with a MDD	A single 25 mg dose	8 weeks	Psilocybin induced a significant reduction in depression severity scores from baseline to posttreatment.
Schindler et al. (2024)	10	Cluster headache	10 mg/70 kg, 3 doses, 5 days apart each	6 months	Psilocybin induced cluster attack frequency was significantly reduced from baseline.
Aaronson et al. (2024)	15	Treatment-resistant bipolar type II major depressive episodes	A single 25 mg dose	12 weeks	12 patients achieved both response and remission after psilocybin administration.
Madsen et al. (2024)	10	Chronic cluster headache	0.14 mg/kg, 3 doses	10 weeks	Attack frequency was reduced by mean 31% from baseline to follow-up after psilocybin administration.
Weiss et al. (2024)	59	MDD	25 mg, 3 doses	6 months	Personality changes were in a direction consistent with improved mental health after psilocybin administration.
Peck et al. (2023)	10	Anorexia nervosa	A single 25 mg dose	1 week	Psilocybin induced signicant reduction in eating disorder-related psychopathology
Lewis et al. (2023)	12	Cancer patients with depression	A single 25 mg dose	26 weeks	Clinically substantial decrease in HAM-D scores after psilocybin administration.
von Rotz et al. (2023)	52	MDD	0.215 mg/kg, 1 dose	2 weeks	Psilocybin induced an absolute decrease in symptom sevetity of −13 points compared to baseline.
Bogenschutz et al. (2022)	95	AUD	25 mg/70 kg (first session) 25-40 mg/70 kg (second session)	36 weeks	Psilocybin induced a decrease in percentage of heavy drinking days.
Gukasyan et al. (2022)	27	MDD	20 mg/70 kg (first session) 30 mg/70 kg (second session)	12 months	Large decrease from baseline in GRID-HAMD scores after psilocybin administration.
Goodwin et al. (2022)	216	TRD	25 mg;10 mg;1 mg (control)	3 weeks	A single dose of 25 mg, but not 10 mg reduced depression scores significantly.
Schindler et al. (2022)	14	Cluster headache	0.413 mg/kg, 3 doses, 5 days apart each	8 weeks	Efficacy outcomes were negative.
Schindler et al. (2021)	12	Migraine	0.413 mg/kg, 2 doses	2 weeks	Significant reducions in migraine measures after psilocybin administration.
Davis et al. (2021)	24	MDD	20 mg/70 kg (first session) 30 mg/70 kg (second session)	4 weeks	Psilocybin increased cognitive and neural flexibility.
Carhart-Harris et al. (2021)	59	MDD	A single 25 mg dose	6 weeks	There was no difference in QIDS-5R-16S depression scores between the psilocybin group and the escitalopram group.
Anderson et al. (2020)	30	Older long-term AIDS survivor men	0.3–0.36 mg/kg, 1 dose	3 months	Demoralization reduced after psilocybin administration.
Stroud et al. (2018)	17	TRD	10 mg (first session) 25 mg (second session)	1 month	Psilocybin improves emotional face recognition.

In further elucidating the pharmacological actions of psilocybin upon additional 5-HT receptors, it becomes paramount to expand our investigative purview to encompass the comprehensive spectrum of psilocybin’s effects, transcending the confines of 5-HT receptors.

### 2.3 Dosage

The optimal dosage of psilocybin continues to pose an unresolved challenge in current research. Conventional investigations rely on a fixed dose ranging from 10 to 30 mg, incorporating micro-dosing or weight-adjusted dosing strategies (Griffiths et al., 2016; Goodwin et al., 2022; Marschall et al., 2022). Nevertheless, recent discoveries indicate that body weight exerts minimal influence on psilocin plasma concentration, and body mass index fails as a predictor of responses to psilocybin, challenging the necessity of weight-adjusted dosing (Holze et al., 2023; Spriggs et al., 2023).

The therapeutic viability of micro-dosing psilocybin for anxiety and depression remains a subject of debate. Some investigations propose that recurrent micro-dosing significantly diminishes negative emotions, amplifies cognitive capabilities (Lea et al., 2020; Rootman et al., 2021, 2022), and offers relief from chronic pain in humans (Lyes et al., 2023). Supporting this notion, there is empirical support indicating that micro-dosing can enhance cognitive functions in rodent models. Noteworthy experiments illustrate that low doses of psilocybin enhance motivation and attention in rats exhibiting suboptimal performance (Higgins et al., 2021). Additionally, micro-dosing with Psilocybin has been demonstrated to ameliorate cognitive deficits observed in a rat model of Fragile X syndrome (Buzzelli et al., 2023). Conversely, alternate findings suggest that while micro-dosing may induce perceptible subjective effects and modify brain electroencephalogram rhythms, no noteworthy distinctions in anxiety, depression, and stress symptoms emerge when compared to a placebo cohort (Cavanna et al., 2022; Marschall et al., 2022). Moreover, at present, there exists a dearth of evidence supporting improvements in happiness, creativity, and cognitive function through micro-dosing in humans (Cavanna et al., 2022; Marschall et al., 2022).

The restricted sample sizes in extant studies contribute to the ambiguity surrounding the optimal dosage of psilocybin and the efficacy of micro-dosing. Consequently, Comprehensive comparative studies encompassing diverse dosage levels are imperative to clarify the optimum psilocybin dose and appraise the effectiveness of micro-dosing.

### 2.4 Gender and/or sex

Earlier investigations hint at the potential for gender variations in the therapeutic outcomes of psilocybin. Notably, the application of psilocybin prompts gender-specific alterations in the reactivity of the central amygdala (Effinger et al., 2023). Moreover, compelling evidence indicates that a singular dose of psilocybin leads to a reduction in ethanol consumption among male C57BL/6 J mice, with no corresponding effect observed in their female counterparts (Alper et al., 2022). These observations underscore the significance of incorporating gender as a crucial biological variable in the context of psilocybin research.

## 3 Potential mechanisms of psilocybin treatment for AD

### 3.1 Neuronal plasticity

#### 3.1.1 Impaired neuroplasticity in AD patients

Neuronal plasticity is an intricate response of neurons to both internal and external stimuli, encompassing a range of mechanisms. These mechanisms include neurogenesis, dendritogenesis, and synaptogenesis (Castrén and Hen, 2013; Von Bernhardi et al., 2017). In the context of AD, autopsy findings have revealed a significant reduction in brain synapses compared to healthy individuals (Chang et al., 2013; Scheff et al., 2016). Positron emission tomography imaging targeting synaptic vesicle glycoprotein 2A (SV2A) has demonstrated a decline in synaptic density in the early stages of AD, which correlates with cognitive function (Mecca et al., 2022). Furthermore, aside from synaptic damage, decreased neurogenesis in the dentate gyrus has been observed, as evidenced by a reduction in the number of doublecortin (DCX)- and bromodeoxyuridine (BrdU)-positive cells in APPswe/PSEN1ΔE9 mice (Arredondo et al., 2021). Importantly, impaired neurogenesis occurs prior to the formation of Aβ plaques and neurofibrillary tangles (Moreno-Jiménez et al., 2019). Overall, these findings shed light on the intricate relationship between neuronal plasticity and the pathological progression of AD.

#### 3.1.2 BDNF/TrkB and mTOR signaling pathways

Neuronal plasticity involves the BDNF/TrkB and mammalian target of rapamycin (mTOR) signaling pathways (Ly et al., 2018). BDNF, a crucial neurotrophic factor, plays important roles in promoting neuronal growth and maturation during development, as well as regulating synaptic transmission and plasticity in adulthood. BDNF and its receptor TrkB are localized at glutamatergic synapses, regulating synaptogenesis, neuronal plasticity, memory formation, and learning (Wang et al., 2022). Deficient BDNF/TrkB activity is associated with neurodegeneration in AD (Wang et al., 2019). As the disease progresses, levels of BDNF decrease in the brains (Aarons et al., 2019), blood (Piancatelli et al., 2022) and cerebrospinal fluid (CSF) of AD patients (Bharani et al., 2020). Deprivation of BDNF/TrkB leads to increased inflammatory cytokines and activates the Janus Kinase 2/Signal Transducer and Activator of Transcription 3 (JAK2/STAT3) pathway. This upregulates the transcription factor CCAAT-enhancer binding protein β (C/EBPβ), resulting in increased expression of δ-secretase. Consequently, both amyloid precursor protein (APP) and Tau are fragmented by δ-secretase, leading to neuronal loss (Wang et al., 2019; Xiang et al., 2019; Wu et al., 2021). Intracerebroventricular injection of adeno-associated viruses encoding human BDNF gene alleviates behavioral deficits, prevents neuronal loss, mitigates synaptic degeneration, and reduces neuronal abnormalities in the brains of P301L mice (Jiao et al., 2016). Furthermore, the mTOR signaling pathway also plays a role in neurogenesis, dendritogenesis, and LTP (Jaworski and Sheng, 2006). The relationship between mTOR signaling and AD is not yet fully understood. Some studies suggest that excessive activation of mTOR signaling in AD patients impairs autophagy, leading to increased Aβ deposition and tau phosphorylation (Rapaka et al., 2022). However, other studies suggest that mTOR activation in microglial cells enhances Aβ plaque clearance in AD animal models.

#### 3.1.3 Psilocybin enhances neuroplasticity

**Figure 3:**
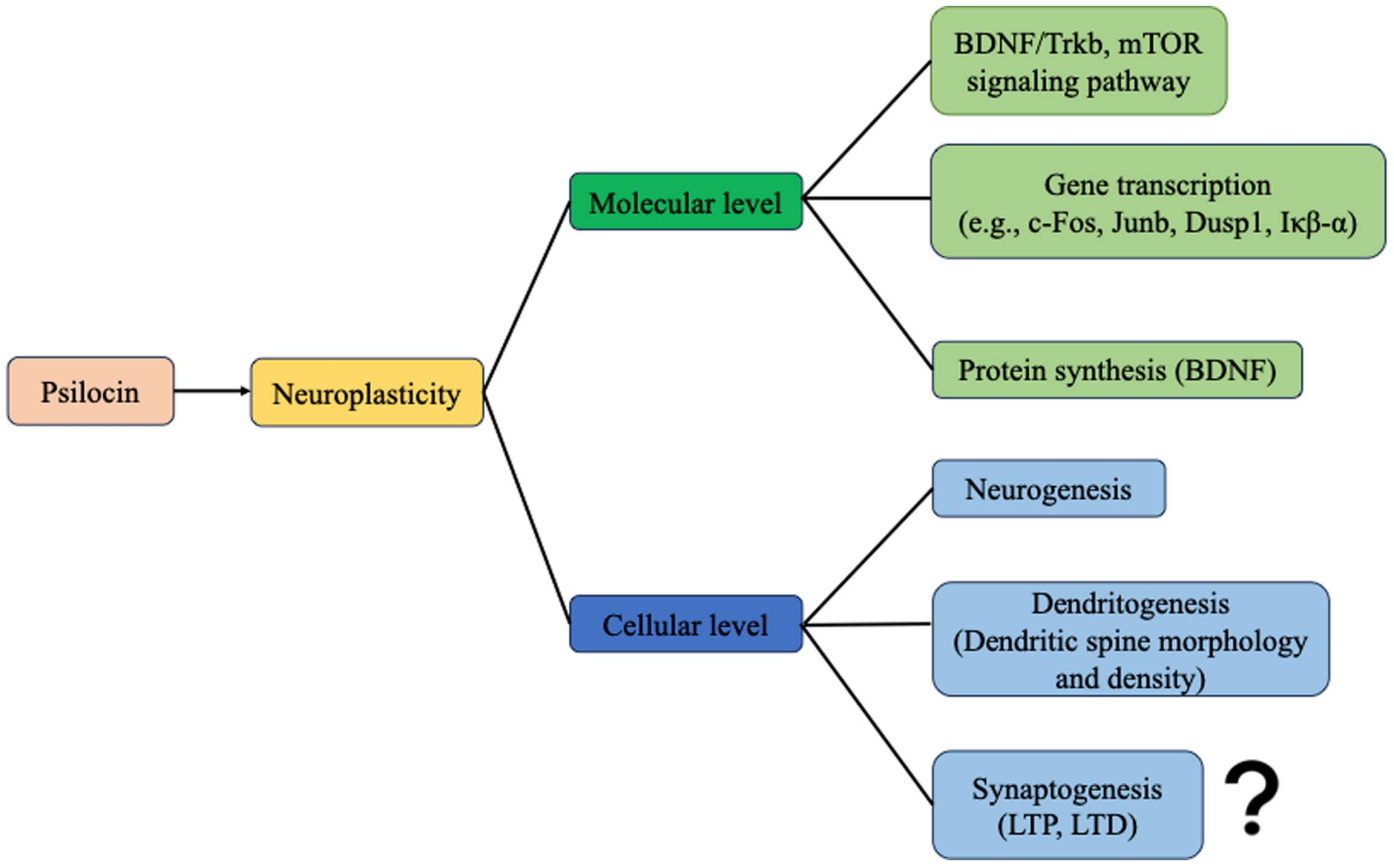
Psilocin promotes neuroplasticity at the molecular and cellular levels. At the molecular level, psilocin enhances neuroplasticity-related signaling pathways, gene transcription, and protein synthesis. And at the cellular level, psilocin promotes neurogenesis and dendritogenesis. However, synaptogenesis has not been evaluated yet.

Psilocybin has demonstrated potential in enhancing neuronal plasticity at both the cellular and molecular levels, suggesting promise for Alzheimer’s disease (AD) treatment (Figure 3). However, it is crucial to acknowledge that much of this remains theoretical or speculative in the absence of robust experimental evidence. Further investigation using AD animal models is necessary to validate these findings.

##### 3.1.3.1 Cellular level

At the cellular level, psilocybin promotes neurogenesis by rescuing the reduction in DCX and BrdU positive cells caused by fear conditioning-induced stress in the hippocampus (Du et al., 2023; Zhao et al., 2024). Moreover, mice administered low doses of psilocybin have shown a swifter extinction of cued fear conditioning compared to those given higher doses. Lower doses of psilocybin are generally correlated with increased neurogenesis (Catlow et al., 2013).

In addition to its neurogenesis-promoting effects, psilocybin also enhances dendritogenesis. For example, it increases the size and density of apical dendritic spines on layer V pyramidal neurons in the medial prefrontal cortex (PFC) by 10%, leading to rapid structural remodeling within 24 h that persists for 1 month (Shao et al., 2021). Similarly, psilocybin rescues the decline in dendritic complexity and spine density induced by conditioned fear in the hippocampus (Du et al., 2023). Additionally, it has been observed to increase the density of SV2A in the hippocampus and PFC of pig brains, indicating greater presynaptic density (Raval et al., 2021). Psilocybin has been shown to mitigate the inhibition of neuroplasticity caused by chronic corticosterone exposure in the PFC and hippocampus. This includes increasing neuroplasticity, as evidenced by a higher total number of dendritic branches and dendritic spine density, as well as elevated levels of synaptic proteins such as phosphorylated GluA1, PSD95, and synapsin-1 (Zhao et al., 2024). Psychedelic mushroom extract and chemically synthesized psilocybin may have different effects on neuroplasticity. Psychedelic mushroom extract significantly increased GAP43, PSD95, synaptophysin, and SV2A across all brain areas, while psilocybin effects were limited to PSD95 and SV2A in the hippocampus and amygdala (Shahar et al., 2024).

LTP and LTD are considered to be fundamental mechanisms underlying synaptic plasticity, which is crucial for learning, memory, and adaptive behavior (Diering and Huganir, 2018). While the effects of psilocybin on the initiation, maintenance, and decline of LTP and LTD have been sparsely explored. Therefore, further research is warranted to enhance our comprehension in this area.

##### 3.1.3.2 Molecular level

**Figure 4:**
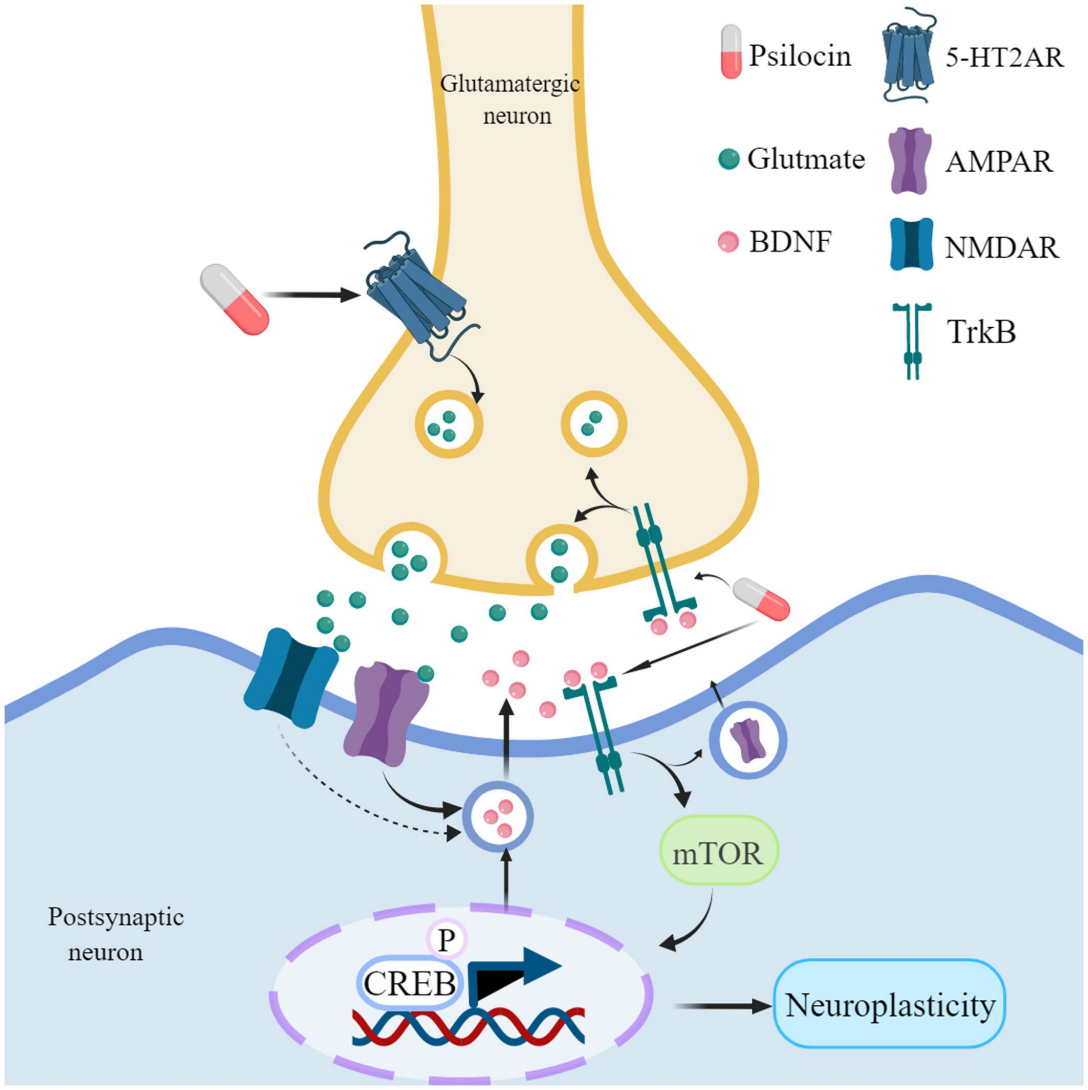
Psilocin promotes neuroplasticity mechanisms. Psilocin interacts with 5-HT2A receptors located on glutamatergic neurons, which in turn promotes the release of glutamate. The release of glutamate can potentially activate both AMPA and NMDA receptors, leading to the release of BDNF. When BDNF binds to its receptor Trkb, it activates the mTOR signaling pathway, resulting in the phosphorylation of CREB. This phosphorylation ultimately leads to an upregulation of genes and proteins associated with neuroplasticity. Furthermore, it facilitates the release of presynaptic glutamate and promotes the transmission of AMPA receptors to the synapse, sustaining the activation of AMPA receptors. In addition, psilocybin may directly bind to TrkB receptors and enhance endogenous BDNF signaling. Created with MedPeer (www.medpeer.cn).

At the molecular level, psilocin primarily exerts its influence on neuronal plasticity by activating the 5-HT2A receptor on glutamatergic pyramidal cells in the deep cortical layers (Figure 4; Vollenweider and Kometer, 2010; Shao et al., 2021). Psilocybin administration leads to a notable increase in extracellular glutamate levels in the prefrontal cortex (Vollenweider and Kometer, 2010). This glutamate release activates α-amino-3-hydroxy-5-methyl-4-isoxazole propionic acid receptor (AMPAR) and N-methyl-D-aspartate receptor (NMDAR) on cortical pyramidal neurons (Vollenweider and Kometer, 2010). However, recent studies have shown that psilocybin administration leads to a significant increase in AMPA current amplitude, while NMDA current amplitude remained unchanged, indicating that psilocybin-induced synaptic enhancement may be regulated by postsynaptic AMPAR activity (Hesselgrave et al., 2021). The binding of glutamate to the AMPAR and NMDAR triggers the release of BDNF, leading to the activation of the BDNF/TrkB pathway (Vollenweider and Kometer, 2010; Zhao et al., 2024).

mTOR regulates the phosphorylation of the cyclic AMP-responsive element-binding protein (CREB), resulting in an upregulation of neuroplasticity-related proteins (including BDNF) and gene expression (e.g., c-Fos, Junb, Dusp1, Iκβ-α) (Takei et al., 2004; Jefsen et al., 2021; Davoudian et al., 2023). Psilocybin increased activity in brain regions associated with stress, reward, and motivation in rats. Specifically, it boosted a protein called Fos in areas like the frontal cortex, nucleus accumbens, amygdala, and locus coeruleus. This effect was stronger with higher doses of psilocybin (Funk et al., 2024). In addition, BDNF can induce the release of glutamate in cortical neurons and enhance the transmission of AMPA receptors to synapses (Takei et al., 1998; Matsumoto et al., 2001; Numakawa et al., 2002; Caldeira et al., 2007). This may explain why AMPAR activation continues even after blocking 5-HT2A receptors with ketaserin. Excessive activation of mTOR can have a dual impact, potentially both promoting synaptic plasticity and impairing autophagy (Jaworski and Sheng, 2006; Rapaka et al., 2022). Therefore, it is crucial to demonstrate that psilocybin does not exacerbate the phenotype in rodent models of AD.

### 3.2 Anti-inflammation

#### 3.2.1 Neuroinflammation and its role in AD progression

Neuroinflammation is of utmost importance in the pathogenesis and advancement of AD. The deposition of Aβ peptides triggers the activation of microglial cells, leading to brain neuroinflammation (Heneka et al., 2013). Activation of the NLRP3 (NOD-, LRR- and pyrin domain-containing 3) inflammasome results in the cleavage and increased activity of caspase-1 within microglial cells, ultimately releasing interleukin (IL)-1β. The tau protein can activate the NLRP3 inflammasome, and the intracerebral injection of brain homogenate containing fibrillar Aβ induces NLRP3-dependent tau pathology changes (Ising et al., 2019). Pro-inflammatory cytokines such as IL-1, IL-6, and tumor necrosis factor alpha (TNF-α) enhance inflammation by stimulating microglial cells and increasing Aβ, thereby contributing to the inflammatory progression of AD. Inflammatory mediators activate cyclooxygenase (COX) -2 in neuroinflammation, which, through the arachidonic acid pathway, synthesizes and releases prostaglandins, further stimulating microglial cells (Dhapola et al., 2021). Triggering receptor expressed on myeloid cells 2 (TREM2), expressed on the surface of microglial cells, has been found to increase the risk of AD when mutated. Genetic ablation of TREM2 resulted in less severe plaque pathology in the early stages of plaque deposition but more severe plaque pathology in the later stages (Carmona et al., 2018). Elevating the amount of TREM2 through the introduction of a human TREM2 transgene reduced pathology in Aβ-bearing mice (Zhong et al., 2019; Wang et al., 2020).

#### 3.2.2 Anti-inflammatory effects of psilocybin

The current research suggests some potential benefits of Psilocybin in the treatment of neuroinflammatory diseases. Activation of the 5-HT2A receptor which is found on lymphocytes, and macrophages, has been proven to regulate immune function and cytokine production (Pellegrino and Bayer, 2002; Xiao et al., 2016). In the context of peripheral immunity, extracts from psilocybin-containing mushrooms have been shown to suppress the production of inflammatory mediators COX-2, nitric oxide, and prostaglandin E2 in Lipopolysaccharide (LPS)-induced macrophages, while also reducing pro-inflammatory cytokines IL-1β, IL-6, and TNF-α, and increasing anti-inflammatory cytokine IL-10 (Nkadimeng et al., 2020a, 2021). Psilocin, but not psilocybin, exerts anti-inflammatory effects on classically activated macrophages (Laabi et al., 2024). Additionally, psilocybin mushrooms have the potential to protect the heart by mitigating TNF-α-induced cardiomyocyte injury and death (Nkadimeng et al., 2020b). Moreover, in an inflammatory response induced by TNF-α and interferon (IFN)-γ in human small intestinal epithelial cells, psilocybin reduces the levels of COX-2 and IL-6 without affecting cell viability. Animal models have also yielded similar results. Psilocybin was shown to reduce inflammation in a 3D model of human intestinal tissue. This anti-inflammatory effect was evidenced by a decrease in TNF-α, IFN-γ, IL-6, and IL-8 levels (Robinson et al., 2023). There is still debate regarding whether psilocybin can alter inflammatory markers in healthy subjects. One study has demonstrated that psilocybin can reduce the concentrations of TNF-α, IL-6, and C-reactive protein (CRP) in healthy volunteers (Mason et al., 2023). However, another research has found no significant changes in the levels of CRP, TNF, or soluble urokinase plasminogen activator receptor (suPAR) (Burmester et al., 2023). It’s possible that the conflicting results could be attributed to the small sample size and the low baseline levels of inflammatory markers in healthy subjects.

Regarding central immunity, psilocybin exhibits significant anti-inflammatory effects in an immortalized microglial cell line, as evidenced by decreased TNF-α secretion in response to LPS challenge (Smedfors et al., 2022). Additionally, psilocin can influence microglial functions by reducing the level of toll-like receptor 4 (TLR4), NF-κB p65 (p65), and cluster of differentiation 80 (CD80) proteins, which are markers of the immune response, and upregulate the TREM2 neuroprotective receptor (Kozłowska et al., 2021). In an LPS-induced mouse brain inflammation model, eugenol, psilocybin, and their combination treatment reduce the expression of various markers such as COX-2, TNF-α, IL-1β, IL-6, and IL-8 (Zanikov et al., 2023). In the 5xFAD mouse model, the activation of mTOR in microglial cells can upregulate TREM2 and promote the clearance of Aβ (Shi et al., 2022). Therefore, it can be speculated that psilocybin may also clear Aβ through this mechanism. Previous research has often focused primarily on peripheral markers when selecting inflammatory markers. Thus, in future studies, it is recommended to enhance the sample size and assess the direct influence of psilocybin on neuroinflammation, for instance, metabolites of the kynurenine-tryptophan, nitric oxide, and neopterin pathways have been suggested as suitable CSF biomarkers for neuroinflammation (Burmester et al., 2023).

The precise anti-inflammatory mechanisms of psilocybin remain to be fully elucidated. While Laabi et al. have proposed potential pathways, further experimental validation is necessary to confirm these hypotheses and gain a comprehensive understanding of psilocybin’s anti-inflammatory actions (Laabi et al., 2024).

Besides, there is also evidence suggesting a connection between autoimmune disorders and AD (Lim et al., 2021). Case studies have indicated that weekly administration of low-dose psilocybin, 3–4 times per week, is effective in treating neuro-Lyme disease. The pathogenesis of neuro-psychiatric Lyme disease is associated with autoimmune-induced neuroinflammation, suggesting that the anti-inflammatory effects of psilocybin may be valuable in autoimmune neuroinflammation (Kinderlehrer, 2023).

### 3.3 Neuropsychology

Neuropsychology constitutes an interdisciplinary field dedicated to elucidating the intricate interplay between the brain and psychology, with a primary focus on the cognitive, emotional, and behavioral functionalities of the cerebral organ. Emerging research suggests that psilocybin may exert effects within the realm of neuropsychology, an area continuously evolving in scientific inquiry and exploration.

#### 3.3.1 Cognitive flexibility

Cognitive flexibility, characterized by the adaptive capacity to transition between diverse cognitive operations in response to changing environmental demands, constitutes a pivotal facet of cognitive function (Uddin, 2021). Evaluation typically involves tasks related to set-shifting or rule-switching, gauging the ability to transition from previously acquired rules (Koch et al., 2018). In rodents, acute administration of psilocybin has been observed to enhance cognitive flexibility, with specificity noted in the augmentation of switching between previously learned behavioral strategies rather than influencing Pavlovian reversal learning. The 5HT 2AR antagonist ketanserin was found to attenuate psilocybin-induced effects on set-shifting, while a 5HT2C-selective antagonist exhibited no such influence (Torrado Pacheco et al., 2023). For healthy adults and individuals with Major Depressive Disorder (MDD), psilocybin treatment has shown promise in augmenting cognitive and neural flexibility (Doss et al., 2021; Nayak et al., 2023).

This enhancement is postulated to stem from increased functional connectivity dynamics between the anterior cingulate cortex and posterior cingulate cortex following psilocybin administration, coupled with the neuroplasticity promoted by the substance (Doss et al., 2021). Additionally, metabotropic glutamate receptor (mGluR) 2 has been implicated in cognitive flexibility. Knocking out neuron-specific frontal mGluR2 in rats resulted in reduced cognitive flexibility and heightened alcohol-seeking behavior, a deficit that psilocybin was able to ameliorate by restoring mGluR2 expression (Meinhardt et al., 2021). Interestingly, mGluR2 is considered a potential target for Alzheimer’s disease (AD) drug treatment. Studies on transgenic mouse models of tauopathy have demonstrated that the selective agonist LY379268, targeting both mGluR2/3, effectively inhibits tau release in synaptosomes and prevents tau propagation between neurons. Conversely, BCI-838, an orally active pro-drug acting as an mGluR2/3 receptor antagonist, has exhibited efficacy in improving learning behavior deficits (Mazzo et al., 2022; Perez-Garcia et al., 2023). Consequently, additional experimental evidence is requisite to elucidate whether the impact of psilocin on mGluR2 could translate into cognitive flexibility improvements.

#### 3.3.2 Emotional face recognition

Mild cognitive impairment (MCI) and AD patients often experience deficits in emotion recognition (Yang et al., 2015; Mazzi et al., 2020). Remarkably, when administered alongside psychological support, psilocybin has shown potential in improving the processing of emotional faces in TRD (Stroud et al., 2018). Furthermore, in studies involving healthy individuals, psilocybin has been observed to act on the 5-HT2A receptor, influencing facial recognition, goal-directed behavior, and emotional states by promoting positive emotions over negative ones (Kometer et al., 2012; Kraehenmann et al., 2015). Patients with TRD or healthy volunteers who receive psilocybin treatment may experience a decrease in amygdala response to negative emotions, likely due to heightened functional connectivity between the amygdala and the ventromedial prefrontal cortex and posterior cingulate cortex. Unlike selective serotonin reuptake inhibitors (SSRIs), which alleviate negative emotions, psilocybin enables patients to confront and address them (Roseman et al., 2018; Barrett et al., 2020; Mertens et al., 2020; Zeifman et al., 2023).

#### 3.3.3 Creativity

Creativity, a fundamental cognitive faculty intricately woven into various facets of daily functioning (Ross et al., 2023), manifests differently in individuals with Alzheimer’s disease (AD), particularly in artistic and literary pursuits. Artists with AD tend to gradually produce paintings characterized by simplicity and abstraction, while writers create works featuring less intricate plots and a constrained vocabulary (Acosta, 2014; Pelowski et al., 2022). A comprehensive examination of psilocybin macrodosing effects on cognition and creativity, elucidated through a scoping review, revealed a time-dependent variation. Initial impairment post-intake was noted, receding over time, with subsequent emergence of positive effects (Bonnieux et al., 2023). However, the controversy surrounding micro-dosing of psilocybin persists. Studies exploring this avenue propose acute enhancements in creativity (Anderson et al., 2019), yet a specific investigation posits that low doses of psilocybe cubensis may not significantly impact creativity (Cavanna et al., 2022). This discrepancy could be attributed to the possibility that the effective dose of psilocybin within psilocybe cubensis might be insufficient to exert a noteworthy influence on creativity.

Psilocybin acutely reduces convergent thinking, increases spontaneous divergent thinking and goal-oriented divergent thinking. These effects are associated with a decrease in the integrity of the default mode network (DMN) and an increase in functional connectivity between resting-state networks induced by psilocybin (Mason et al., 2019, 2021).

### 3.4 AD comorbid with depression and anxiety

#### 3.4.1 The acceleration of AD progression by depression and anxiety

Depression and anxiety are prevalent neuropsychiatric manifestations in individuals with AD. Intriguingly, a discernible genetic correlation between depression and AD has been identified, suggesting a shared genetic foundation (Martín-Sánchez et al., 2021; Monereo-Sánchez et al., 2021; Harerimana et al., 2022). Noteworthy is the association of late-onset depression and anxiety with a significantly elevated risk of dementia development, with the severity of depression being linked to an increased susceptibility to AD. Moreover, anxiety has been implicated in the deposition of Aβ protein and cognitive decline (Becker et al., 2018; Chan et al., 2020; Johansson et al., 2020; Kuring et al., 2020; Kim D et al., 2021; Kim H et al., 2021). It is crucial to emphasize that depression and anxiety serve not only as risk factors for AD but also actively contribute to the progression of the disease (Gallagher et al., 2018; Lee et al., 2019; Ruthirakuhan et al., 2019; Ma, 2020; Agüera-Ortiz et al., 2021).

#### 3.4.2 Psilocybin therapy for depression and anxiety in AD

There is currently no consensus regarding the preferred choice of treatment for depression in patients with dementia, with selective serotonin reuptake inhibitors (SSRIs) appearing to be one commonly considered option. Initial findings from a longitudinal study suggested that the use of antidepressants may result in a slower decline in cognition and daily functioning (Dutcher et al., 2014). However, the Health Technology Assessment Study of the Use of Antidepressants for Depression in Dementia (HTA-SADD) trial contradicted this, revealing no therapeutic efficacy of sertraline or mirtazapine and an increased risk of adverse events (Banerjee et al., 2013; Zuidersma et al., 2019). A recent systematic review and meta-analysis of randomized controlled trials failed to demonstrate any beneficial effects of second-generation antidepressants on cognition and depression, including various informative subgroups (Qin et al., 2022). Another systematic review and network meta-analysis indicated psychoeducation was the sole effective intervention against anxiety (Sun et al., 2022). Consequently, the management of depression in individuals with Alzheimer’s disease (AD) and comorbid anxiety necessitates innovative therapeutic approaches.

Notably, an open-label pilot study initiated by Johns Hopkins University in 2019 aims to assess the safety and efficacy of administering psilocybin under supportive conditions for depression in patients with mild cognitive impairment (MCI) or early-stage AD. Preliminary results from this study are expected to be disclosed in 2024 (ClinicalTrials.gov NCT04123314).

Addressing the symptoms of depression and anxiety accompanying Alzheimer’s disease (AD) becomes crucial for slowing down disease progression. Several clinical studies have presented evidence supporting the effectiveness of psilocybin in treating MDD (Carhart-Harris et al., 2016, 2021; Davis et al., 2021), TRD (Goodwin et al., 2022, 2023a), and depression and anxiety symptoms in advanced cancer patients (Griffiths et al., 2016).

The DMN refers to a group of brain regions that show decreased activation during goal-directed or attention-demanding tasks. It primarily includes the medial PFC, posterior cingulate/retrosplenial cortex, and left and right inferior parietal lobules (Whitfield-Gabrieli and Ford, 2012). Individuals with anxiety and depression share similar alterations in the DMN, characterized by increased functional connectivity within the DMN and reduced connectivity between the DMN and other higher-order networks like the salience network and central executive network (Whitfield-Gabrieli and Ford, 2012; Li et al., 2023). In comparison to escitalopram, psilocybin exhibits a faster onset, better tolerance, and longer duration of effect, potentially leading to improved outcomes. Psilocybin may contribute to a decrease in functional connectivity within the DMN and an increase in connectivity between the DMN and other functional networks rich in 5-HT2A receptors (Carhart-Harris et al., 2021; Daws et al., 2022; Gukasyan et al., 2022; Zeifman et al., 2023). Combining psilocybin with mindfulness meditation and other psychological therapy techniques could be beneficial, as both mindfulness and psilocybin therapy seem to modulate the DMN in similar ways and may have a synergistic effect (Smigielski et al., 2019). Additionally, psilocybin treatment has been associated with reduced amygdala cerebral blood flow, correlating with the alleviation of depressive symptoms (Carhart-Harris et al., 2017). The decrease in amygdala blood flow caused by psilocybin may reflect the neuroregulatory effects of 5-HT1A receptors (Lewis et al., 2017). Furthermore, transient elevation of plasma glucocorticoids supports psilocybin-induced anxiolysis in mice, suggesting that acute, resolvable psilocybin-induced glucocorticoid release drives the post-acute anxiolytic-like effects of psilocybin in mice (Jones et al., 2023).

## 4 Adverse effect

Clinical investigations spanning from 2018 to the present, emphasizing the utilization of psilocybin in the treatment of Major Depressive Disorder (MDD), Treatment-Resistant Depression (TRD), migraine, and other maladies, alongside trials involving subjects without pre-existing conditions, reveal a low incidence of severe adverse events. Predominantly reported adverse reactions encompass transient and self-limiting manifestations such as headache, nausea, vomiting, fatigue, among others (Table 2). These reactions are often dose-dependent and typically do not necessitate specific interventions (Davis et al., 2021; Schindler et al., 2021; Perez et al., 2023). Symptomatic medications like paracetamol or ibuprofen can be employed, if required, to mitigate headaches (Goodwin et al., 2023b). Notably, a clinical trial reported serious adverse events including suicidal ideation, codeine withdrawal syndrome, and self-injury depression (Goodwin et al., 2022).

**Table 2:** Side effects of psilocybin (clinical trials of psilocybin published from 2018-present).

References	Size	Subjects	Adverse events								
			Headache/migraine	Nausea/vomiting	Fatigue	Anxiety/depression	Visual illusion/hallucination	Hypertension	Tension/sore muscles	Abdominal pain/bloating/diarrhea	Palpitations/ventricular tachycardia
Ley et al. (2023)	32	Healthy participants	✓	✓	✓	✓		✓		✓	✓
Peck et al. (2023)	10	Anorexia nervosa	✓	✓	✓						
Lewis et al. (2023)	12	Cancer patients with depression	✓	✓				✓			✓
von Rotz et al. (2023)	52	MDD	✓	✓						✓	
Rucker et al. (2022)	89	Healthy participants	✓		✓		✓				
Bogenschutz et al. (2022)	95	AUD	✓	✓		✓				✓	✓
Gukasyan et al. (2022)	27	MDD	✓				✓				
Goodwin et al. (2022)	216	TRD	✓	✓	✓						
Becker et al. (2022)	23	Healthy participants	✓	✓	✓					✓	
Schindler et al. (2022)	14	Cluster headache	✓	✓	✓	✓			✓	✓	
Holze et al. (2022)	28	Healthy participants	✓	✓				✓			
Schindler et al. (2021)	12	Migraine	✓						✓		
Davis et al. (2021)	24	MDD	✓			✓					✓
Carhart-Harris et al. (2021)	59	MDD	✓	✓	✓					✓	✓
Anderson et al. (2020)	30	Older long-term AIDS survivor men	✓	✓		✓	✓	✓			✓
Stroud et al. (2018)	17	TRD	✓		✓	✓					

In the current examination of psilocybin’s adverse reactions, several limitations merit acknowledgment. Firstly, the modest sample size in some studies may lead to the omission of infrequent adverse events, thus constraining the generalizability of the results. Secondly, the studied patient population often excludes individuals with a history of mental illness and severe suicide attempts. Administering psilocybin to such patients without rigorous supervision could result in severe consequences, exemplified by a case report detailing a manic episode following psilocybin use in an individual with bipolar II disorder (Daws et al., 2022; Gukasyan, 2023; Halim et al., 2023). Thirdly, the scarcity of placebo-controlled trials hinders the establishment of a causal relationship between psilocybin and adverse events. Numerous variables, encompassing drug dosage, age, gender, education, and experimental conditions, may influence adverse reactions in patients. Fourthly, as psilocybin trials broaden to include a larger participant pool, it becomes imperative to investigate whether the increase in blood pressure induced by psilocybin has implications for hypertension prevalence in the general population. Additionally, uncertainties persist regarding potential significant adverse reactions arising from the combination of psilocybin with other psychotropic medications, necessitating exploration into the advisability of discontinuing these medications before undergoing psilocybin treatment.

The administration of psilocybin requires rigorous supervision, given the potential serious consequences of self-administration. Several case reports underscore the risks associated with self-administration of psilocybin mushrooms, including takotsubo cardiomyopathy (Nef et al., 2009; Kotts et al., 2022), severe rhabdomyolysis (Bickel et al., 2005; Suleiman et al., 2022), bipolar disorder (Hendin and Penn, 2021), distressing flashback phenomena hallucinogen (Müller et al., 2022), and even persisting perception disorder (Espiard et al., 2005).

## 5 Removing the hallucinogenic effects of psilocybin

### 5.1 Hallucinogenic effects constrain the development of psilocybin

Profound investigations into the mechanisms of psilocybin’s action are imperative for addressing its hallucinogenic effects. The distinctive psychedelic properties of psilocybin raise concerns about its prospective clinical application. Effective mitigation of its hallucinogenic effects is paramount for potential clinical utilization, even upon prospective market approval. Importantly, it should be noted that hallucination is not classified as a side effect.

A pivotal inquiry emerges: Are the hallucinogenic effects of psilocybin inherently therapeutic, or does their separation result in a decline in therapeutic efficacy? The current absence of non-hallucinogenic 5-HT2A agonists with demonstrated clinical effectiveness in humans underscores the imperative for further investigation. Additionally, scrutinizing the limitations of existing non-hallucinogenic antidepressants is essential, particularly considering the reliance on rodent models and tests that may lack direct applicability to humans.

### 5.2 Exploring methods to reduce the hallucinogenic effects of psilocybin

**Figure 5:**
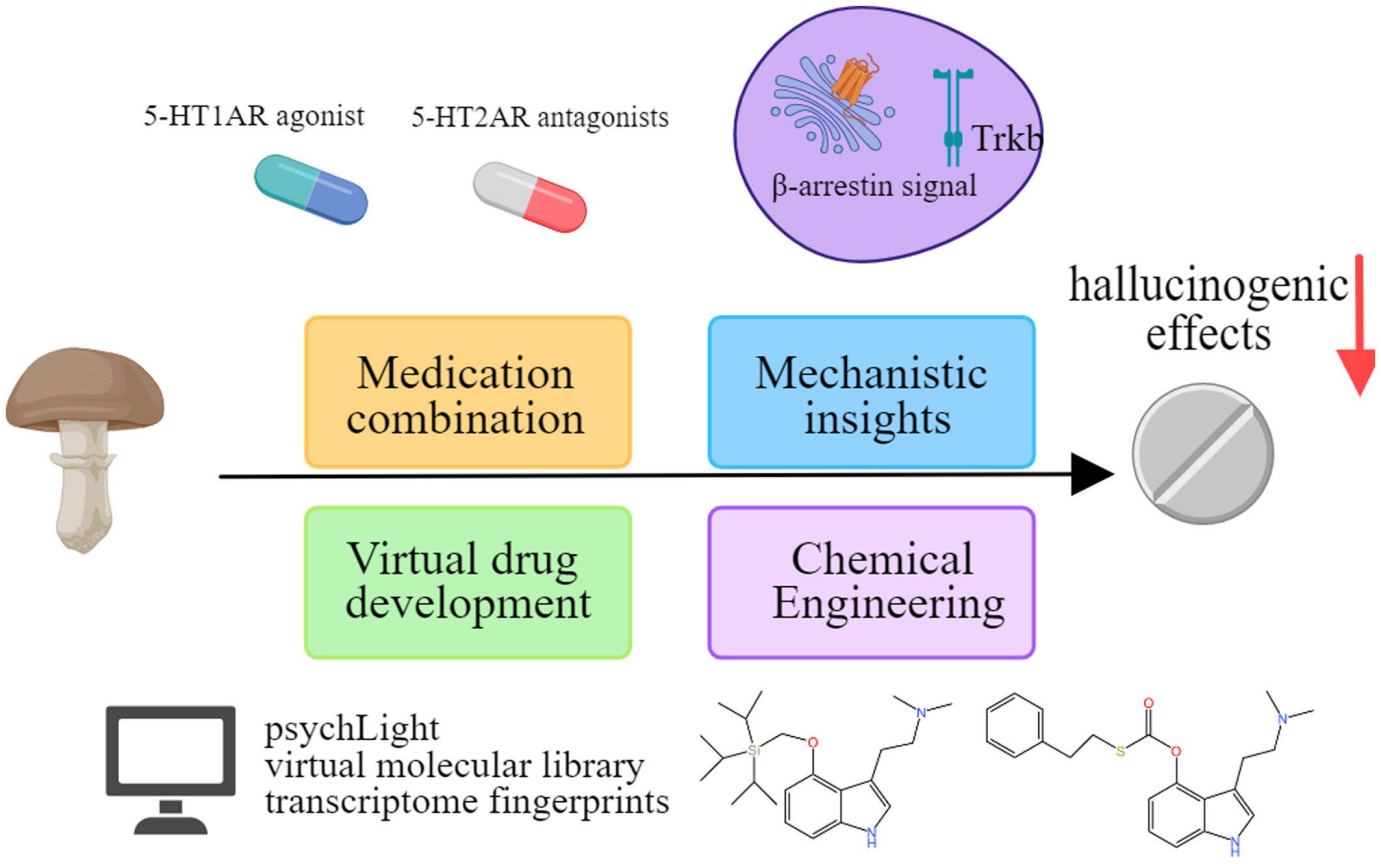
Exploring methods to reduce the hallucinogenic effects of psilocybin.

Several approaches are explored to reduce psilocybin’s hallucinogenic effects (Figure 5).

#### 5.2.1 Medication combination

The head twitch response mediated by the 5-HT2A receptor, triggered by psilocybin, is observed to be reduced by the agonist activity of the 5-HT1A receptor, exemplified by buspirone, especially at elevated doses in mice (Glatfelter et al., 2023; Singh et al., 2023). Despite the potential inhibitory effect of 5-HT2A receptor antagonists on the head twitching reaction, caution is warranted, as blocking the 5-HT2A receptor may significantly impact therapeutic efficacy. Given the plausible involvement of the 5-HT2A receptor in mediating neural plasticity, blocking 5-HT2A receptor may not be an optimal choice.

#### 5.2.2 Mechanistic insights

In-depth research into the mechanisms of psilocybin’s action contributes to discerning its hallucinogenic effects. Although both psilocin and serotonin interact with the 5-HT2A receptor, the specific neuroplasticity-promoting effects observed with psilocybin are not exhibited by serotonin. This distinction is primarily attributed to a location bias, as the intracellular 5-HT2A receptor mediates the neuronal plasticity induced by psilocin, rather than the receptor on the cell membrane (Vargas et al., 2023). In addition to the classical binding mode of psilocin to 5-HT2A receptor, there is an alternative lipid-regulated binding mode that plays a crucial role in activating β-arrestin signaling downstream of 5-HT2A receptor through agonist bias. With this understanding, researchers have designed and synthesized compounds like IHCH-7086 and IHCH-7079, demonstrating rapid antidepressant effects without hallucinogenic side effects in animal experiments (Cao et al., 2022). Research has also indicated the pursuit of structure-based design methodologies for developing high-affinity TrkB-selective ligands characterized by swift and enduring antidepressant effects, while concurrently exhibiting a potential absence of hallucinogenic-like activity (Moliner et al., 2023). Although some studies speculate that the activation of Gs proteins plays a key role in distinguishing between hallucinogenic and non-hallucinogenic 5-HT2A receptor agonists (Liu et al., 2022), this viewpoint may be intricate, considering that the interaction between 5-HT2A receptor and Gs proteins might be just one of many factors influencing hallucinogenic properties.

#### 5.2.3 Virtual drug development

Recent advancements in computer drug development technology have transformed the concept of “virtual drugs” into reality (Gorgulla et al., 2020). The utilization of psychLight, a gene-encoded fluorescence sensor based on the structure of the 5-HT2A receptor, enables precise prediction of the psychedelic behavioral effects of structurally similar ligands that target the 5-HT2A receptor. Moreover, it also facilitates the identification of non-psychedelic analogs that exhibit antidepressant properties (Dong et al., 2021). By exploring the virtual molecular library and discovering a molecule that shares similar characteristics to psilocybin, followed by a comparison of its 3D structure with that of 5-HT2A receptor, it becomes feasible to synthesize a candidate compound that possesses antidepressant activity without any hallucinogenic effects (Kaplan et al., 2022). In addition, transcriptome fingerprints have proven successful in distinguishing between the hallucinogenic and nonhallucinogenic effects of 5-HT2A receptor agonists in mice (González-Maeso et al., 2003). These findings contribute to a rational design approach for the development of psilocybin analogs targeting specific signal pathways.

#### 5.2.4 Chemical engineering

Through precise chemical engineering, researchers are actively working to modify psilocybin, aiming to produce safer variants retaining therapeutic efficacy while eliminating hallucinogenic properties. In recent studies, researchers have introduced a diverse range of cleavable groups at the 4-hydroxy position of the core indole moiety. This strategic modification aims to modulate metabolic processing, resulting in the creation of a novel prodrug of psilocin. Among these active prodrugs, two distinct molecules have demonstrated lasting anxiolytic benefits in chronically stressed mice, as evaluated in the marble-burying psychiatric model (Raithatha et al., 2023).

In general, these methods require in-depth research and clinical trials to ensure that the modified drugs meet the expected standards in terms of safety and therapeutic efficacy.

## 6 Legal and ethical issues

Psilocybin remains a substance of significant controversy, eliciting varied societal perspectives on its utilization (Doss et al., 2022). In light of this, ensuring the legal and ethical recognition of psilocybin treatment for AD is paramount.

### 6.1 Legality

Psilocybin encountered pervasive regulatory constraints within the realm of clinical psychiatry commencing in 1967, concomitant with its legal reclassification as a Schedule I controlled substance. This classification limited its usage and research potential. This categorization precipitated limitations on both its clinical utilization and research prospects. In the year 2015, a treatise featured in the British Medical Journal articulated the imperative for a legal reclassification of psychedelic substances, positing a compelling argument for researchers to delve into their therapeutic potential (Rucker, 2015).

Evolving regulatory landscapes have witnessed a gradual relaxation of restrictions pertaining to the medical applications of psilocybin in several nations. In the United States, Psilocybin presently occupies the classification of a Schedule I controlled substance according to federal mandates. Despite this federal designation, select states have commenced authorizing its medical deployment. A watershed moment occurred in May 2019, as Denver, Colorado emerged as the inaugural U.S. city to decriminalize psilocybin, while 2020 saw Oregon becoming the pioneering U.S. state to legalize its supervised medical application for the amelioration of mental disorders (Basky, 2021; Siegel et al., 2023). Projections posit a trajectory wherein the majority of U.S. states will have legislatively embraced psychedelics by the temporal juncture spanning 2034–2037 (Siegel et al., 2023). Besides, Canada has been progressively expanding avenues for therapeutic access to psychedelic substances. In the year 2020, historic exemptions were granted, permitting legal possession and personal use of psilocybin mushrooms—signifying a landmark development nearly five decades subsequent to their criminalization (Mocanu et al., 2022). Recent developments in Jamaica reveal a paradigm shift, where adult patients within psychiatric practice settings have been presented with the option of incorporating psilocybin products into their treatment regimens for conditions encompassing major depressive disorder, post-traumatic stress disorder, substance use disorders, and existential distress (De La Haye et al., 2023). On the antipodean front, noteworthy endeavors have been undertaken in Australia to pave the way for the prescription of MDMA and psilocybin in the treatment of post-traumatic stress disorder and depression—an epochal stride within the global landscape (Haridy, 2023).

However, a prevailing disposition persists in which the majority of countries or regions continue to regard psilocybin as an illicit substance. Divergent perspectives on legalization manifest across societies and nations, underscored by historical, cultural, and legal differentials. It is incumbent upon researchers and practitioners to exercise due diligence within the bounds of legal and regulatory frameworks, ensuring strict adherence to pertinent legislations.

### 6.2 Informed consent

In the context of conducting psilocybin research on AD patients, the informed consent process demands particular attention and meticulous consideration. Given the cognitive decline faced by AD patients, ensuring the effectiveness of the informed consent process is paramount for safeguarding patient rights (Gauthier et al., 2013). Prior to the commencement of the study, a rigorous individual assessment, specifically focusing on cognitive capabilities, is imperative. If significant impairment in cognitive capacity is identified among AD patients, researchers must ascertain the necessity of legal guardians’ involvement and ensure their legal eligibility for providing informed consent (Lawrence et al., 2014; Ortiz et al., 2022).

The materials provided to patients for informed consent should be lucid, succinct, and tailored to the patient’s level of comprehension, presented through both oral and written modalities. Given the novel therapeutic process, mechanism, and potential unexpected side effects of psilocybin, including shifts in values and personality, rare mental health side effects, and the potential use of therapeutic touch during therapy, special attention is warranted in the context of enhanced consent (Smith and Sisti, 2020).

Considering the potential memory issues faced by AD patients, the informed consent process may necessitate multiple iterations (High, 1992). Repeatedly confirming consent at different time points ensures that patients maintain a clear understanding and possess the ability to withdraw consent at any given moment during their participation in the study. Despite the fluctuating cognitive states of AD patients, their right to decline participation in research should be consistently respected.

### 6.3 Patient safety and well-being

During the administration of Psilocybin, ensuring a systematic safety monitoring protocol is paramount, encompassing the surveillance of physiological metrics and mental states. Similarly, the formulation of contingency plans is imperative to address potential adverse reactions or unforeseen incidents (Belouin et al., 2022). Given the absence of prior Psilocybin utilization in AD patients, it is crucial, during Psilocybin administration, to ensure the presence of a mental health therapist or a qualified psychological professional for support. These professionals should be equipped to navigate and address potential emotional or cognitive responses that may arise during Psilocybin use.

### 6.4 Appropriate experimental design

Given the potential variability in individual responses to Psilocybin, particularly among AD patients, a precise calculation of personalized doses is imperative. Furthermore, the duration of Psilocybin administration and the overall research protocol should be individualized to cater to the unique needs of each patient.

The cognitive state of AD patients may influence their perception of time and processing abilities (Honma et al., 2015). Therefore, the study design should contemplate the reduction of experimental duration to accommodate potential shorter attention spans and tolerance levels in patients (Ortiz et al., 2022).

Given that the psychedelic properties of Psilocybin may induce fatigue or emotional fluctuations in patients (Griffiths et al., 2011), the research design should allow for adequate resting intervals. This approach aids in alleviating the psychological burden on patients and facilitates adaptive periods.

In the context of AD patients, consideration should be given to involving family members or caregivers. Their participation can provide supplementary support, assist in monitoring patient responses, and offer additional safety and emotional backing when necessary.

## 7 Conclusions and perspectives

Recent strides in utilizing psilocybin for the treatment of neuropsychiatric disorders exhibit considerable promise. Psilocybin holds potential for conferring distinctive advantages in terms of neuroprotection and cognitive enhancement for individuals with Alzheimer’s disease (AD) through mechanisms such as neuroplasticity, inflammation regulation, and enhanced neuropsychology. Furthermore, the antidepressant and anti-anxiety effects of psilocybin may signify substantial advancements in addressing the mental well-being of patients with neuropsychiatric conditions associated with AD. Nonetheless, it is imperative to recognize that a substantial journey lies ahead before psilocybin can be judiciously and efficaciously employed in AD patient care. Stringent scientific inquiry is indispensable to comprehensively discern its efficacy, safety profile, and optimal utilization. Further exploration is warranted to elucidate the precise mechanisms of action of psilocybin. Moreover, the viability of alternative strategies, including combination therapies involving psilocybin, the development of high-affinity TrkB positive allosteric modulators without 5-HT2A activity, and modification of psilocybin’s chemical composition to obviate hallucinogenic effects, necessitates investigation. Additionally, regulatory approvals and ethical considerations demand meticulous attention prior to embarking on clinical trials. Prudent development of psilocybin-based treatments for AD mandates adherence to rigorous scientific standards and ethical guidelines.

## Author contributions

SZ: Writing – original draft, Writing – review & editing. RM: Conceptualization, Supervision, Writing – original draft. YY: Conceptualization, Supervision, Writing – original draft. GL: Conceptualization, Supervision, Writing – review & editing.

## Figures and Tables

**Table:** 

AD	Alzheimer’s disease
APP	Amyloid precursor protein
Aβ	Amyloid-beta
AUD	Alcohol use disorder
AMPAR	α-amino-3-hydroxy-5-methyl-4-isoxazole propionic acid receptor
BDNF	Brain-derived neurotrophic factor
BrdU	Bromodeoxyuridine
CD80	Cluster of differentiation 80
C/EBPβ	CCAAT-enhancer binding protein β
CREB	Cyclic AMP-responsive element-binding protein
CRP	C-reactive protein
CSF	Cerebrospinal fluid
DCX	Doublecortin
DMN	Default mode network
FDA	The U.S. Food and Drug Administration
5-HT2A receptor	5-hydroxytryptamine 2A receptor
IL	Interleukin
IFN	Interferon
JAK2/STAT3	Janus Kinase 2/Signal Transducer and Activator of Transcription 3
LPS	Lipopolysaccharide
LTD	Long-term depression
LTP	Long-term potentiation
MAO	Monoamine oxidase
MDD	Major depressive disorder
mGluR	Metabotropic glutamate receptor
mTOR	Mammalian target of rapamycin
NMDAR	N-methyl-D-aspartate receptor
p65	NF-κB p65
PFC	Prefrontal cortex
suPAR	Soluble urokinase plasminogen activator receptor
SSRIs	Selective serotonin reuptake inhibitors
SV2A	Synaptic vesicle glycoprotein 2A
TLR4	Toll-like receptor 4
TNF-α	Tumor necrosis factor alpha
TrkB	Tropomyosin receptor kinase B
TRD	Treatment-resistant depression
Trem2	Triggering receptor expressed on myeloid cells 2
UGT	UDP-glucuronosyltransferases

## Footnotes

^1^https://pdsp.unc.edu/databases/kidb.php
